# Breakthrough of glycobiology in the 21st century

**DOI:** 10.3389/fimmu.2022.1071360

**Published:** 2023-01-05

**Authors:** Gehendra Mahara, Cuihong Tian, Xiaojia Xu, Jinxiu Zhu

**Affiliations:** ^1^ Clinical Research Center, The First Affiliated Hospital of Shantou University Medical College, Shantou, Guangdong, China; ^2^ Department of Cardiovascular Medicine, The First Affiliated Hospital of Shantou University Medical College, Shantou, Guangdong, China; ^3^ Center for Precision Health, Edith Cowan University, Perth, WA, Australia; ^4^ Department of Infection Control, The First Affiliated Hospital of Shantou University Medical College, Shantou, Guangdong, China; ^5^ Institute of Clinical Electrocardiography, The First Affiliated Hospital of Shantou University Medical College, Shantou, Guangdong, China; ^6^ Longgang Maternity and Child Institute of Shantou University Medical College, Shenzhen, Guangdong, China

**Keywords:** glycobiology, glycan, carbohydrate, nanotechnology, drug development, vaccine

## Abstract

As modern medicine began to emerge at the turn of the 20th century, glycan-based therapies advanced. DNA- and protein-centered therapies became widely available. The research and development of structurally defined carbohydrates have led to new tools and methods that have sparked interest in the therapeutic applications of glycans. One of the latest omics disciplines to emerge in the contemporary post-genomics age is glycomics. In addition, to providing hope for patients and people with different health conditions through a deeper understanding of the mechanisms of common complex diseases, this new specialty in system sciences has much to offer to communities involved in the development of diagnostics and therapeutics in medicine and life sciences.This review focuses on recent developments that have pushed glycan-based therapies into the spotlight in medicine and the technologies powering these initiatives, which we can take as the most significant success of the 21st century.

## Glycobiology and glycomics

Over the past few decades, the use of computational modeling in the field of glycobiology has grown due to the rising popularity of glycobiology and glycomics (1). One of the newest omics disciplines to emerge in the contemporary post-genomics age is glycomics. In addition, to providing hope for patients and people with different health conditions through a deeper understanding of the mechanisms of common complex diseases, this new specialty in system sciences has much to offer to communities involved in the development of diagnostics and therapeutics in medicine and life sciences (2). Glycomics is a branch of glycobiology that focuses on defining the structure and function of glycans in living organisms. Glycobiology is the study that focuses on the structure, function, and biology of carbohydrates (1, 3). An emerging discipline is known as “systems glycobiology (the impact of systems biology on the study of glycome),” which is hopeful given the current availability of advancing Wet-Lab techniques in the fields of glycobiology and glycomics (4).

A long chain of carbohydrate-based polymers called glycans is made up of repeating monosaccharide monomer units joined by glycosidic connections. All cells in nature appear to contain complex and varied glycans, which are crucial to all biological systems. In living things, glycans play physical, structural, and metabolic roles (4). The last century appeared to be a significant expansion in our understanding of the biochemistry and biology of proteins and nucleic acids (5, 6).

## Genomics revolution and biotechnology

Scientific interest in understanding the characterization, function, and interaction of other essential biomolecules such as DNA transcripts, proteins, lipids, and glycans for the cell has grown due to the genomics revolution and the advent of high-throughput technologies (7). High-throughput technology enables the production of massive amounts of data for omic analysis, for example, genomics, transcriptomics, proteomics, phenomics, and metabolomics (8). At present, the growth of these technologies and their application go hand-in-hand with the growth of bioinformatics (9). Glycopeptide-based antibiotics (GBA) such as Vancomycin, Teicoplanin, Telavancin, Ramoplanin, and Decaplanin, including Corbomycin, Complestatin, and antitumor antibiotic Bleomycin, are another breakthrough invention of this century (10). Two blockbuster drugs, Acarbose (Bayer) and Heparin, including influenza treatment drugs Tamiflu (Oseltamivir, Roche) stand out as monosaccharide-based drugs that have been used therapeutically for a long time and saving the lives of people (11, 12).

Studies on glycobiology have grown as high-throughput technologies have advanced, allowing for fast cell screening. Additionally, more sophisticated analytical methods and data processing tools offer the chance to enhance high-throughput approaches for glycan screening as a disease marker and for categorizing glycan structure in therapeutic proteins (13). In addition, nanotechnology is a process of modifying matter at a size close to the atomic level to create a novel structure, materials, and devices. This technique offers advances in science across a wide range of industries, including manufacturing, consumer goods, energy, transportation, food safety, environmental science, and medicine, among many others (14).

## Glyco-nanoparticles and nanotechnology

In addition, nanoparticles (NPs) are currently gaining a lot of attention due to their use in biology and medicine. The primary biological applications include the identification, estimation separation, purification, and characterization of biological molecules and cells, as well as the use of fluorescent biological labels, MRI contrast enhancement agents, pathogen and protein detection, DNA probing, tissue engineering applications, tumor targeting, targeted delivery of drugs, genes, and small molecules (15).

The development of powerful tools with diagnostic, therapeutic, and analytical applications through the use of nanotechnology has changed the approach of biomedical sciences and fight against human diseases (16). Millions of lives have been saved annually from vaccination, which is a success story in global health and development. More than 20 deadly diseases (such as Polio, Tetanus, Flu (influenza), Hepatitis B and Hepatitis A, Rubella, Hib, Measles, Whooping Cough, Pneumococcal, Rotavirus, Mumps, Chickenpox, Diphtheria, and off course end of 2021, COVID-19 and so on) can now be saved by vaccines, allowing individuals of all ages to live longer (17, 18). The milestone intervention has been created in medical history by developing the vaccine for cancer, such as the HPV (human papillomavirus) vaccine, to prevent cervical, vaginal, and vulvar cancer, anal cancer, and genital warts used for oral cancer. Likewise, the Hepatitis B vaccine treats existing liver cancer (also called therapeutic vaccine-immunotherapy) (12, 19).

Nanotechnology focuses on hybrid materials made of inorganic nanostructures and biomolecules (20–22). Synthetic scaffolds made of iron oxide, noble metal, and semiconductor nanoparticles have been used to multimerize glycans and increase their affinity for receptors. Hybrid material’s physical features, such as magnetic and fluorescence, have led to applications in sensing, delivery, and imaging, e.g., ultraviolet-visible (UV-Vis) spectroscopy, infrared (IR) spectroscopy, elemental analysis, nuclear magnetic resonance (NMR), transmission electron microscopy (TEM), and X-ray photoelectron spectroscopy (XPS) (23). Likewise, as contrast agents for magnetic resonance imaging (MRI), magnetic nanoparticles (MNPs), such as iron oxide and manganese oxide nanoparticles (MONPs), are of particular interest. MRI uses a radio frequency (RF)-induced electromagnetic field to generate internal tomographic tissue pictures; modification of that field’s signal by particles (called “contrast”) allows their location to be noticed. Targeted magnetic, photodynamic, and gene therapy have all been used to battle cancer using nanocarriers based on heparin and heparin derivatives (24). Multifunctional gold NPs (nanoparticles) containing polysaccharide-functionalized gold NPs have been developed in various applications, including imaging, photodynamic treatment, and apoptosis activation of metastatic cells (24, 25). Likewise, heparin’s capacity to inhibit blood clot formation has enabled significant medical advancements since world war-II, such as heart transplants, renal dialysis, and coronary artery dilations (angioplasties) (26).

## Glyco-science and therapeutics uses

In recent decades, various functions of glycans in biological systems have been discovered due to the growing glycoscience study. Numerous scientific fields, including immunology, development and differentiation, biopharmaceuticals, cancer, fertility, blood types, infectious illnesses, etc., have identified significant roles of glycans” (9). Glycan receptors are being targeted to treat viral diseases. The antiviral drugs Zanamivir (Relenza) and Oseltamivir (Tamiflu) are perhaps the most successful sugar-based medications these days. Likewise, carbohydrate-based antivirus medicines are Remedesivir, Molnupiravir, Azvudine, Entecavir, Telbivudine, Clevudine, Sofosbuvir, and Maribavir; those drugs are competitive neuraminidase ligands that bind to the enzyme and prevent the virus particle from being released from host cells (16, 27).

Due to their diversity, glycans have a wide range of biological functions and play essential roles in many physiological and pathological processes, including cell division, differentiation, and tumour formation (28, 29). Glycans are essential biomarker candidates for many diseases, including cardiovascular diseases, immune system deficits, genetically inherited disorders, various cancer types, and neurological diseases, carry information in biological system (30–33). During the onset and progression of these disorders, altered glycan expression is seen, brought on by improperly controlled enzymes, including glycosyltransferases and glycosidases. As a result, altered glycan structures may be helpful in the early detection of certain disorders. Glycans play an important role in illness diagnosis and management. Still, they can also be employed therapeutically as markers to identify and isolate particular cell types and as targets for developing new medications (3, 34).

## Geoinformatics databases and glycosylation

Numerous biological processes, such as cell growth and development, tumour growth and metastasis, immunological detection and response, cell-to-cell communication, and microbial pathogenesis, are significantly influenced by glycosylation. Due to course, one of the most prevalent and critical posttranslational modifications of proteins is glycosylation (35, 36). Several factors can influence and modify glycosylation, including genetic determinants, monosaccharide nucleotide levels, cytokines, metabolites, hormones, and ecological factors (35–39). To get a large picture of the entire biological system, it is crucial to integrate omics methods such as proteomics, genomics, transcriptomics, and metabolomics into the field of glycobiology (35, 36, 40). Additionally, a wide variety of geoinformatics resources and databases are now available to investigate glycans and glycosylation pathways, which is also one breakthrough invention of the century (13).

## Chromatography, diagnostic and therapeutic

Several methods have been developed and used in recent years to determine the structure of glycans to various degrees of detail (41). A conventional approach involves radioactively labelling the glycoconjugates, followed by enzymatic or chemical treatments, anionic exchange, gel filtration, or paper chromatographic analysis. Studies using nuclear magnetic resonance (NMR), gas chromatography with mass spectrometry (GC-MS), and other methods were carried out extensively. In recent years, simple chromatography methods have been replaced by High-performance anion-exchange chromatography (HPLC) and Ultra-performance liquid chromatography (UPLC), and fluorescence labelling has taken the role of radioactive labelling. Chromatography columns can be utilized in conjunction with the proper enzymatic/chemical treatments, for example, graphitized carbon, reversed-phase (RP), anion exchange, normal phase, or hydrophilic interaction resins (9, 42). The most precise and widely used separation and purification method is column chromatography. Column chromatography can be used to separate and purify both solid and liquid materials. The extraction of pesticides of animals origin (made up of lipids, waxes, and pigments) has been aided by column chromatography. The chromatography process is used in medicine to create the peptide hormone pramlintide (an analog of amylin), which is used to treat diabetes and many more (43).Various glycan detection technologies have shed light on the nature of several diseases, including COVID-19, diabetes, cancer, and congenital abnormalities (44–46).

Numerous diseases that afflict humans are treated, cured, or even prevented using information from DNA. Researchers have already worked on gene sequencing to find specific genes that cause diseases, allowing them to develop remedies. The development of biomedicine has been greatly aided by gene therapy. The health community and the general public believe the human genome draft sequencing will enable researchers to provide cures or at least effective therapies for all ailments (47).

A more detailed and exact understanding of skin ageing is made possible by the recent improvement in glycobiology, which has been made possible by cutting-edge technological advancements. The field of longevity and anti-aging has been revolutionizing by the anti-ageing healthcare technology (48, 49). Cutting-edge technology is the use of the latest and most advanced version of technology or applications that make function easy, cost-effective, reliable, and fast. Cutting-edge technology can be software that is also regarded as a “game changer”. The cloud, containers, AI (artificial intelligence), and machine learning are all considered cutting-edge technologies (50). Patient care services, chronic disease management, and patient health initiatives, including rapid virus testing during COVID-19, digital diagnostics, telehealth, drug delivery, vaccine development, skin grafting, cancer and diabetics treatment and modern, etc., are examples of applications (51, 52). Glycans are now undeniably proven to be essential skin elements and play a critical function in skin homeostasis. Glycans, which are essential for skin health, also change qualitatively and quantitatively as we age (53).

## Conclusion

Since the beginning of modern medicine in the last century, the genomics revolution, geoinformatics database, biotechnology, and development in chromatology including the progress of glycan-based therapies have advanced rapidly. Research and development of structurally defined carbohydrates have led to the use of new tools and methods that have fueled interest in the therapeutic applications of glycans. DNA- and protein-centered therapies became widely used and progressed toward success. However, more precise targets for glycomimetics need to be found. Studying complex glycosylated structures, particularly glycoproteins, has advanced significant developments in synthetic procedures, analytical tools, and high-resolution biophysical approaches in glycobiology. Nanoparticles and other polyvalent structures have been developed to improve specially formulated glycopeptides’ avidity and therapeutic potential, which can be considered the biggest success of the 21st century.

## Author contributions

GM was responsible for the concept, design and draft of the manuscript. CT, XX contributed to the collect the information and reviewed this manuscript. JZ reviewed the final version of the manuscript and approved by all authors. All authors contributed to the article and approved the submitted version.
